# Substrate-induced condensation activates plant TIR domain proteins

**DOI:** 10.1038/s41586-024-07183-9

**Published:** 2024-03-13

**Authors:** Wen Song, Li Liu, Dongli Yu, Hanna Bernardy, Jan Jirschitzka, Shijia Huang, Aolin Jia, Wictoria Jemielniak, Julia Acker, Henriette Laessle, Junli Wang, Qiaochu Shen, Weijie Chen, Pilong Li, Jane E. Parker, Zhifu Han, Paul Schulze-Lefert, Jijie Chai

**Affiliations:** 1 State Key Laboratory of Plant Environmental Resilience, College of Biological Sciences, China Agricultural University, Beijing, China; 2 Department of Plant Microbe Interactions, Max Planck Institute for Plant Breeding Research, Cologne, Germany; 3 Institute of Biochemistry, University of Cologne, Cologne, Germany; 4 Key Laboratory of Cell Proliferation and Regulation Biology, Ministry of Education, Beijing Key Laboratory of Gene Resource and Molecular Development, College of Life Sciences, Beijing Normal University, Beijing, China; 5 Dana-Farber Cancer Institute, Harvard Medical School, Howard Hughes Medical Institute, Boston, MA USA; 6 School of Life Sciences, Westlake University, Institute of Biology, Westlake Institute for Advanced Study, Hangzhou, Zhejiang China; 7 Tsinghua–Peking Joint Center for Life Sciences, Center for Plant Biology, School of Life Sciences, Tsinghua University, Beijing, China; 8 Cluster of Excellence on Plant Sciences, Max Planck Institute for Plant Breeding Research, Cologne, Germany; 9 Present Address: Cluster of Excellence on Plant Sciences, Max Planck Institute for Plant Breeding Research, Cologne, Germany

**Keywords:** Plant immunity, Enzyme mechanisms, Cell death, Plant signalling

## Abstract

Plant nucleotide-binding leucine-rich repeat (NLR) immune receptors with an N-terminal Toll/interleukin-1 receptor (TIR) domain mediate recognition of strain-specific pathogen effectors, typically via their C-terminal ligand-sensing domains^1^. Effector binding enables TIR-encoded enzymatic activities that are required for TIR–NLR (TNL)-mediated immunity^2,3^. Many truncated TNL proteins lack effector-sensing domains but retain similar enzymatic and immune activities^4,5^. The mechanism underlying the activation of these TIR domain proteins remain unclear. Here we show that binding of the TIR substrates NAD^+^ and ATP induces phase separation of TIR domain proteins in vitro. A similar condensation occurs with a TIR domain protein expressed via its native promoter in response to pathogen inoculation in planta. The formation of TIR condensates is mediated by conserved self-association interfaces and a predicted intrinsically disordered loop region of TIRs. Mutations that disrupt TIR condensates impair the cell death activity of TIR domain proteins. Our data reveal phase separation as a mechanism for the activation of TIR domain proteins and provide insight into substrate-induced autonomous activation of TIR signalling to confer plant immunity.

## Abstract (web summary)

Binding of the substrates NAD^+^ and ATP to the plant Toll/interleukin-1 receptor (TIR) domain proteins induces phase separation and, thereby, activation of TIR enzymatic and immune signalling activity.

## Main

**Extended Data Fig. 1:**
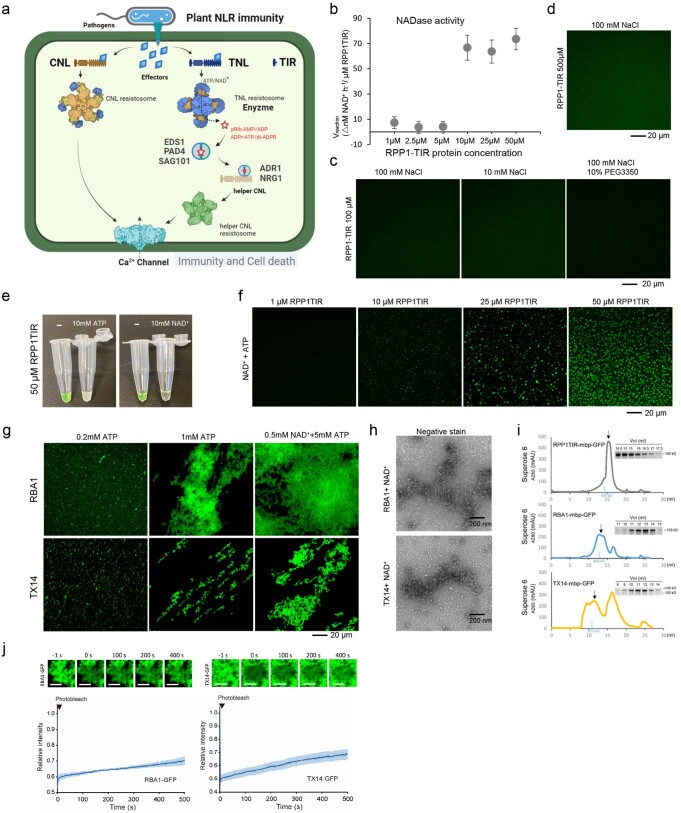
NAD^+^/ATP induces phase separation of TIR domain proteins in vitro. **a**, A schematic of the NLR immunity signaling pathway. Pathogen effectors induce activation of plant NLRs, leading to the formation of oligomeric NLR complexes termed resistosomes. Activation of a CNL forms a pentameric CNL resistosome, which directly targets the cell membrane as a Ca^2+^ channel and mediates Ca^2+^ influx to trigger immune response and cell death. Activation of a TNL forms a tetrameric TNL resistosome, which acts as NADase holoenzyme to catalyze production of small molecules pRib-AMP/ADP and ADPr-ATP/di-ADPR. The small immune molecules bind to and allosterically activate lipase-like EDS1 and its paralog partners PAD4 and SAG101. The activated EDS1-PAD4 and EDS1-SAG101 heterodimers respectively induce assembly of helper CNL ADR1 and NRG1 resistosomes as Ca^2+^ channels, leading to immune response and cell death. The cartoon images were created with BioRender.com. **b**, NADase activity of the RPP1-TIR protein. The NADase reactions were performed in 25 °C in the presence of 100 μM NAD^+^ and 10 mM MgCl_2_ for 24 h. Data are mean ± SD (n = 3 biologically independent samples). **c** and **d**, RPP1-TIR has no phase separation activity in low-salt, in the presence of PEG, or high protein concentrations in the absence of NAD^+^ or ATP. The protein concentrations and buffer conditions were indicated. Scale bar = 20 μm. **e**, ATP or NAD^+^ (10 mM, final concentration) induced RPP1-TIR protein (50 μM) turbidity within 10 s. **f**, NAD^+^/ATP induced phase separation of the RPP1-TIR protein. NAD^+^/ATP with physiological concentration (0.5 mM/5 mM) were incubated with the RPP1-TIR protein with different concentrations at 25 °C for 30 min. Scale bar = 20 μm. **g**, ATP and NAD^+^/ATP induced phase separation of the TIR domain protein RBA1 and TX14. ATP or NAD^+^/ATP with different concentrations were incubated with 10 μM RBA1 and TX14 at 25 °C for 30 min. Scale bar = 20 μm. **h**, Negative staining electron microscopy images showing that RBA1 or TX14 form high-order assembly structures in the presence of 1 mM NAD^+^. Scale bar = 200 nm. **i**, Gel-filtration assay of purified GFP-fused RPP1-TIR, RBA1, and TX14 proteins. The elution peaks were indicated with arrows. The peak fractions were visualized using SDS-PAGE followed by Coomassie blue staining. The molecular weight markers of Superose 6 column were indicated in gel-filtration profiles. **j**, Dynamics analysis of RBA1 and TX14 aggregates by FRAP. Time 0 s indicates the time of the photobleaching pulse. Scale bar = 20 μm. The experiments in **c**, **d** and **f**–**j** were repeated at least three times with similar results. Source Data

The perception of non-self molecules by the innate immune system of plants is mediated largely by two types of immune receptors^1^. One type is cell membrane-localized pattern recognition receptors (PRRs). PRRs perceive features of microorganisms that are often conserved among widely related taxa in the extracellular space to elicit basal immunity, also referred to as pattern-triggered immunity^6^ (PTI). The second type is intracellular NLR receptors, which detect microorganism effectors inside plant cells to confer effector-triggered and pathogen strain-specific immunity^7^ (ETI). Activation of ETI results in termination of pathogen growth and often a localized, hypersensitive host cell death response at sites of attempted pathogen invasion^7^. Mounting evidence in *Arabidopsis thaliana* supports a crosstalk between PTI and ETI, which potentiates the immune response^8–11^. Pathogen-detecting NLRs are divided into two main classes according to their N-terminal domains: coiled-coil NLRs (CNLs) and TNLs^12^. Upon recognition of pathogen effectors, CNLs form resistosomes (pathogen-activated NLR oligomers) that can function as Ca^2+^-permeable channels^13,14^. By contrast, effector binding to the C-terminal domains of TNLs induces the formation of tetrameric TNL resistosomes, enabling their TIR-encoded NADase activity^2–5^. TNL resistosomes have an additional ADP-ribosylation activity^15^. The NADase and ribosyl-transferase activities of TIRs catalyse the production of small molecules which bind to and allosterically activate dimers of the lipase-like protein ENHANCED DISEASE SUSCEPTIBILITY 1 (EDS1) and its direct partners PHYTOALEXIN DEFICIENT 4 (PAD4) or SENESCENCE-ASSOCIATED GENE 101 (SAG101)^15,16^. Once activated, EDS1–PAD4 and EDS1–SAG101 dimers interact directly with downstream CNL-type helper NLRs, ACTIVATED DISEASE RESISTANCE 1 (ADR1) and N REQUIREMENT GENE 1 (NRG1), respectively, which presumably activates their Ca^2+^-permeable channel activity to mediate disease resistance and cell death^15–18^ (Extended Data Fig. 1a).

In addition to canonical TNLs, plant genomes encode many truncated TNLs that lack the C-terminal effector-sensing domains^19,20^. In particular, monocotyledonous plants have TIR-only proteins but not TNLs^21^. Transient gene expression of such TIR domain proteins in *Nicotiana* *benthamiana* can be sufficient to trigger NADase-dependent cell death^4,20,22,23^, suggesting that TNLs and TIRs share conserved signalling pathways. For example, the TIR-only protein RBA1 in *A. thaliana* accession Ag-0 responds to the bacterial pathogen effector HopBA1 to trigger *EDS1*-dependent ETI^23^. Self-association mediated by conserved interfaces is important for the NADase and immune activities of TIRs^23,24^. As well as ETI, TIR signalling also has a role in PTI and abiotic stress responses^10,11,25^. PTI elicitors induce activation of TIR signalling, which in turn boosts the PTI response^10,11^, whereas pathogen effector binding is required to stimulate the enzymatic activities of TNLs^2,3^. TIR domain proteins lacking a ligand-sensing domain are often transcriptionally induced in response to pathogen stimuli and enzymatically activated even in the absence of a pathogen effector^4,5,10^. The underlying mechanism of TIR activation remains largely unknown.

Here we provide evidence that plant TIR domain proteins form substrate-induced condensates in vitro and in planta. Disruption of condensation compromises NADase and cell death activities of TIR domain proteins. Our results reveal a mechanism that underpins the activation of TIR domain proteins via phase separation, and provide insights into substrate-induced autonomous activation of TIR signalling.

## Substrates induce phase separation of TIR

**Fig. 1:**
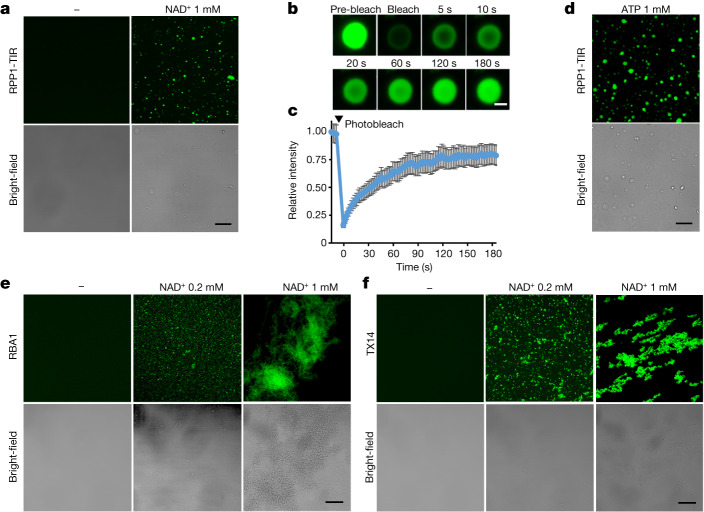
NAD^+^/ATP induces phase separation of TIR-only proteins in vitro. **a**, Images showing NAD^+^-induced liquid-like droplets of RPP1-TIR–GFP. NAD^+^ (1 mM) was incubated with 25 μM RPP1-TIR protein in the presence of 5% polyethylene glycol (PEG) 3,350 at 25 °C for 30 min. Scale bar, 10 μm. **b**, Dynamics analysis of RPP1-TIR–GFP droplets by FRAP. The photobleaching pulse ends at 0 s. Scale bar, 1 μm. **c**, Quantification of the RPP1-TIR–GFP droplets in FRAP assays. Data are mean ± s.d. (*n* = 3 biologically independent samples). **d**, Images showing ATP-induced droplets of RPP1-TIR–GFP; 1 mM ATP was incubated with 25 μM RPP1-TIR protein in the presence of 5% PEG 3,350 at 25 °C for 30 min. Scale bars, 10 μm. **e**,**f**, NAD^+^ induces liquid-to-solid transition of the RBA1 and TX14 TIR domain proteins. NAD^+^ was incubated with 10 μM RBA1 or TX14 at 25 °C for 30 min. Scale bars, 20 μm. **a**,**d**,**e**,**f**, Experiments were repeated at least three times with similar results. Source Data

The NADase activity of plant TIR domain proteins requires relatively high protein concentrations^2,5^. Consistent with this idea, we found that the NADase activity of the TIR domain of RPP1 (RPP1-TIR) was markedly increased at a concentration above 10 μM (Extended Data Fig. 1b). This non-linear kinetics of RPP1-TIR NADase activity suggested a concentration-dependent enzymatic activity, which prompted us to explore whether a TIR domain protein has phase-separation activity^26,27^. To test this possibility, we purified recombinant RPP1-TIR–GFP and examined its phase-separation activity at a higher concentration (100 μM), in the presence of a crowding agent, and in a low-salt condition, conditions that are known to promote phase separation or electrostatic modulated phase separation^28^. Confocal microscopy analyses showed no phase separation of RPP1-TIR–GFP protein under these conditions (Extended Data Fig. 1c). Even at 500 μM, we did not observe phase separation of RPP1-TIR–GFP (Extended Data Fig. 1d). Substrate- or ligand-induced phase separation has been observed for many proteins^29,30^. Indeed, addition of the substrate NAD^+^ induced the formation of many liquid-like droplets of RPP1-TIR–GFP at 25 μM (Fig. 1a). Fluorescence recovery after photobleaching (FRAP) assays showed that the RPP1-TIR–GFP droplets displayed fluorescence recovery within seconds, indicating a highly dynamic internal environment of these droplets (Fig. 1b,c). Since ATP is also a substrate for TIR-catalysed products^15^, we tested whether it also induces phase separation of the RPP1-TIR–GFP protein. As anticipated, ATP induced phase separation of the RPP1-TIR–GFP protein with even higher phase-separation activity than NAD^+^ (Fig. 1d, Extended Data Fig. 1e and Supplementary Video 1). Simultaneous addition of NAD^+^ and ATP^31,32^ (NAD^+^/ATP) (at physiological concentrations, 0.5 mM NAD^+^ and 5 mM ATP) also efficiently induced RPP1-TIR–GFP phase separation (Extended Data Fig. 1f). Together, these results show that the RPP1-TIR protein undergoes phase separation that is triggered by NAD^+^/ATP in vitro.

We then investigated whether phase-separation activity of RPP1-TIR is conserved in TIR-only proteins. We purified recombinant GFP-fused TIR-only proteins RBA1–GFP and TX14–GFP because of their established role in plant immune responses^10,23,33^. RBA1 mediates *Arabidopsis* accession-specific immunity in response to the pathogen effector HopBA1 delivered by *Pseudomonas syringae*^23^, whereas TX14 (AT2G32140) is a PTI-induced TIR domain protein^10^ that mediates *EDS1*-dependent autoimmunity and defence-related gene expression when over-expressed in *Arabidopsis*^20,33^. Similar to RPP1-TIR–GFP, RBA1–GFP and TX14–GFP underwent phase separation in the presence of NAD^+^/ATP (Fig. 1e,f and Extended Data Fig. 1g). However, in contrast to the liquid-like droplets of RPP1-TIR–GFP, RBA1–GFP and TX14–GFP formed larger aggregate-like structures at physiological concentrations of NAD^+^/ATP (Fig. 1e,f and Extended Data Fig. 1f). Negative-staining electron microscopy further confirmed the presence of these aggregate-like structures (Extended Data Fig. 1h). One possible reason for the difference in the condensates is that RBA1 and TX14 have a stronger ability to self-associate than RPP1-TIR (Extended Data Fig. 1i). Consistent with this notion, FRAP assays showed relatively low dynamics of RBA1–GFP and TX14–GFP, suggesting that RBA1–GFP and TX14–GFP proteins form gel-like condensates (Extended Data Fig. 1j). The features of the RBA1 and TX14 condensates are reminiscent of liquid-to-solid phase separation-induced *oskar* ribonucleoprotein granules^34^. In conclusion, our data indicate that several tested plant TIR domain proteins exhibit NAD^+^/ATP-induced phase-separation activity in vitro.

## Multi-interfaces mediate TIR condensation

**Fig. 2:**
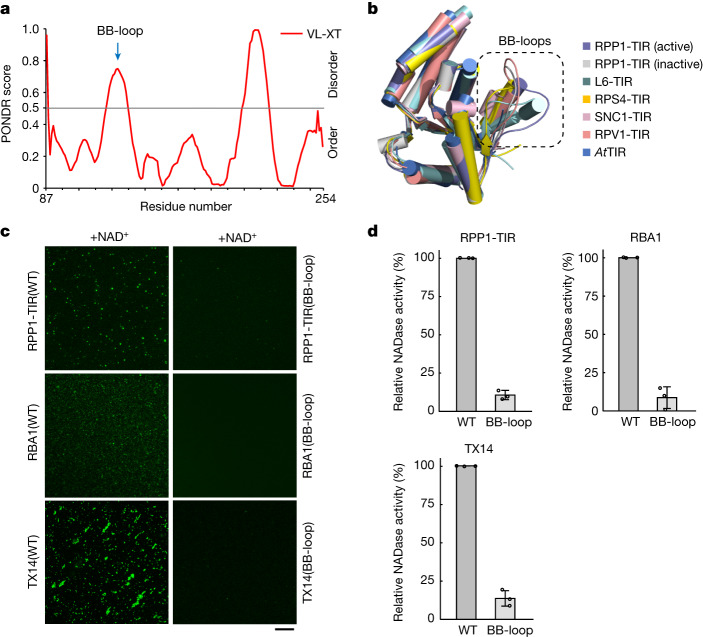
Multiple interfaces mediate phase separation of TIR domain proteins. **a**, Prediction of IDRs in RPP1-TIR by the PONDR VL-XT algorithm. The BB-loop region is indicated. **b**, Structural alignment of TIR domain proteins. BB-loops are highlighted. **c**, BB-loop mutations disrupt phase separation of TIR domain proteins. 1 mM NAD^+^ was incubated with 25 μM RPP1-TIR(WT) or RPP1-TIR(BB-loop) protein in the presence of 5% PEG 3,350 at 25 °C for 30 min; 0.2 mM NAD^+^ was incubated with 10 μM RBA1(WT), RBA1(BB-loop), TX14(WT) or TX14(BB-loop) proteins at 25 °C for 30 min. Scale bar, 20 μm. The experiments were repeated at least three times with similar results. **d**, Relative NADase activity of wild-type and BB-loop mutant RPP1-TIR, RBA1 and TX14 proteins. The NADase activity of wild-type TIR domain proteins was normalized to 100%. Data are mean ± s.d. (*n* = 3 biologically independent samples). Source Data

**Extended Data Fig. 2:**
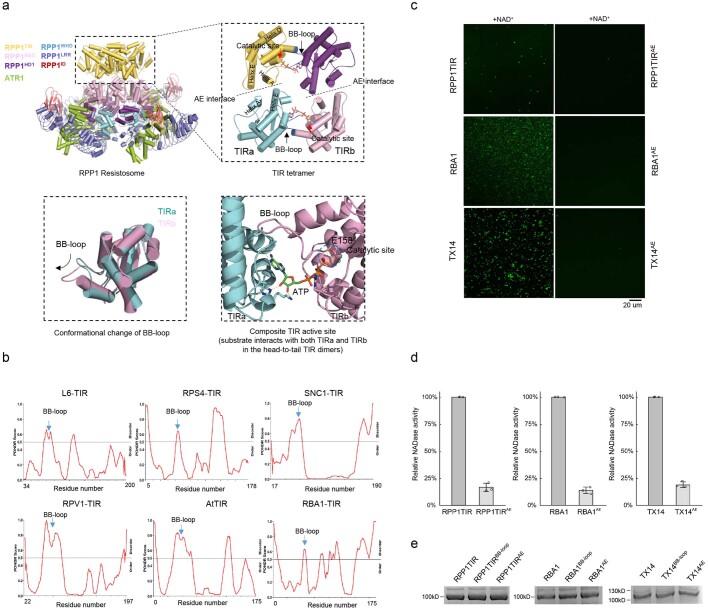
BB-loop and AE interface are important for phase separation of TIR domain proteins. **a**, A schematic of the enzymatic active TIR tetramer of the RPP1 resistosome. The catalytic site, BB-loop, AE interface and helices A, D, and E are labeled. The conformational change of BB-loop and ATP interaction with head-to-tail TIR dimer are highlighted. **b**, IDR prediction of TIR domain proteins by PONDR® server. **c**, AE interface mutations disrupt phase separation of TIR domain proteins. 1 mM NAD^+^ was incubated with 25 μM RPP1-TIR^WT^ or RPP1-TIR^S102D^ protein in the presence of 5% PEG 3,350 at 25 °C for 30 min. 0.2 mM NAD^+^ was incubated with 10 μM RBA1^WT^, RBA1^AE^, TX14^WT^, or TX14^AE^ proteins at 25 C for 30 min. Scale bars = 20 μm. The experiments were repeated at least three times with similar results. **d**, Relative NADase activity of WT and AE mutant, RPP1-TIR, RBA1, and TX14 proteins. The NADase activity of WT TIR domain protein was normalized to 100%. Data are mean ± SD (n = 3 biologically independent samples). The concentrations of TIR proteins used for reactions were 40 μM. **e**, The input of the RPP1-TIR, RBA1, and TX14 variant proteins used for NADase assays. The experiments were repeated at least three times with similar results. Source Data

We next probed the mechanism underlying the phase-separation activity of TIR domain proteins. Conformational changes in the TIR catalytic site caused by substrate binding may be important for TIR phase separation induced by substrates. Formation of composite active TIR NADase catalytic centres in the head-to-tail TIR dimers of the TNL resistosomes involves conformational change in a loop region called the BB-loop^2,3^ (Extended Data Fig. 2a). Sequence-based predictions indicated that the BB-loop is a potential intrinsically disordered region (IDR) in RPP1-TIR and other TIR domain proteins (Fig. 2a and Extended Data Fig. 2b). This is further confirmed by several crystal structures of TIR domains^2,24,35–37^, which consistently show a more flexible BB-loop region compared with other parts of these proteins (Fig. 2b). To provide experimental evidence for this model, we generated mutations of RPP1-TIR–GFP by substituting the polar and charged residues from the BB-loop with alanine residues E122A/R123A/S124A/K125A/S126A (RPP1-TIR(BB-loop)). Confocal microscopy data showed that the phase-separation activity of RPP1-TIR was greatly reduced by the BB-loop mutations (Fig. 2c). We then tested whether the BB-loop regions of RBA1 and TX14 have a similar role in their phase-separation activity. Similar mutations were made in the BB-loops of RBA1 (R52A/G53A/N54A/D55A (RBA1(BB-loop)))–GFP and TX14 (E57A/V58A/R59A/G60A/K61A/D62A (TX14(BB-loop)))–GFP. The resulting RBA1(BB-loop) and TX14(BB-loop) were greatly compromised in their phase-separation activity (Fig. 2c). These results suggest a conserved role of the BB-loop in driving phase separation of TIR domain proteins. To investigate whether phase separation of TIR domain proteins is associated with their enzymatic activity, we measured the NADase activity of wild-type and BB-loop mutants of these three TIR proteins using a previously established assay^2^. Whereas wild-type RPP1-TIR, RBA1 and TX14 proteins nearly completely consumed the NAD^+^ substrate, NAD^+^-hydrolysing activity was significantly reduced in the corresponding BB-loop mutant proteins (RPP1-TIR(BB-loop), RBA1(BB-loop) and TX14(BB-loop)) (Fig. 2d).

Multivalent interactions are important for phase separation of macromolecules^38^. Indeed, plant TIR domains are known to have multiple functional self-association interfaces^24^, including the AE interface, which is formed by helices αA and αE (Extended Data Fig. 2a). Because this interface is important for self-association of all TIR domain proteins tested^24,35^, we reasoned that it might also be involved in TIR phase separation. To test this hypothesis, mutated residues at the predicted AE interface of RPP1-TIR (S102D (RPP1-TIR(AE)))–GFP, RBA1 (S31A/H32A (RBA1(AE)))–GFP and TX14 (S38D (TX14(AE)))–GFP and assayed the phase-separation activity of the resulting mutant proteins. The results showed that RPP1-TIR(AE)–GFP, RBA1(AE)–GFP and TX14(AE)–GFP mutant proteins lost phase-separation activity in vitro (Extended Data Fig. 2c). In further support of previous data^4,23,24^, the mutations of the AE interface impaired the NADase activity of these TIR domain proteins (Extended Data Fig. 2d,e).

## Condensation in TIR-mediated cell death

**Fig. 3:**
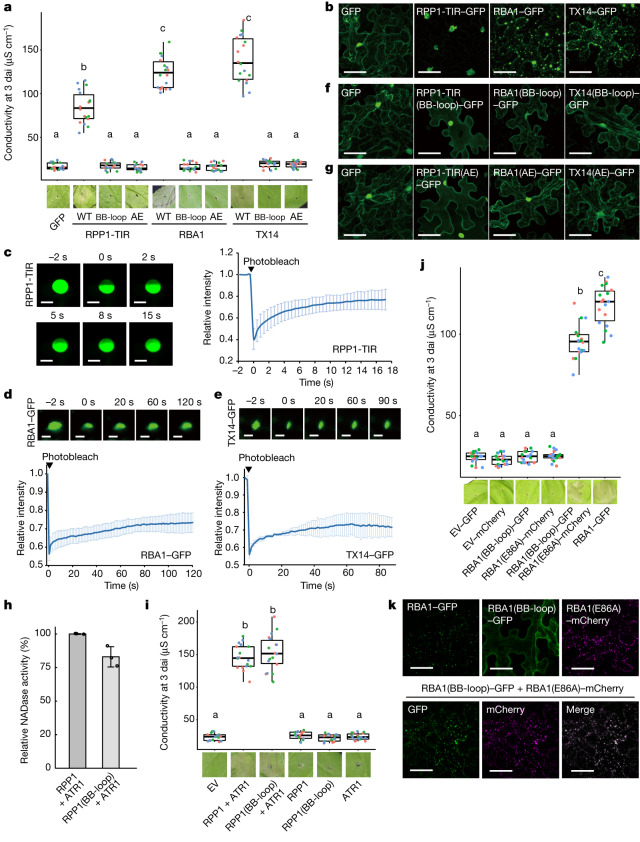
Phase separation is essential for TIR-triggered cell death in *N. benthamiana*. **a**,**i**,**j**, Cell death phenotype of *N. benthamiana* transiently expressing C-terminally GFP-tagged RPP1-TIR, RBA1 and TX14 variants (**a**), full-length RPP1 and RPP1(BB-loop) with or without the cognate effector ATR1 (**i**), or RBA1–GFP, RBA1(BB-loop)–GFP, RBA1(E86A)–mCherry and co-expressing RBA1(BB-loop)–GFP or RBA1(E86A)–mCherry (**j**). Top, ion-leakage assay at 3 days after agro-infiltration (dai). Results from 3 independent experiments are shown (*n* = 18 biologically independent samples; analysed by one-way ANOVA with Tukey’s honest significant difference (HSD) test, *P* = 0.001). In box plots, the centre line indicates the median, the bounds of the box show the 25th and the 75th percentiles, and whiskers indicate 1.5× the inter-quartile range (IQR). Bottom, images of representative leaf zones after agro-infiltration at 4 dai. Groups labelled with the same letter are not significantly different from each other. **b**,**f**,**g**, Confocal images of *N. benthamiana* transiently expressing C-terminally GFP-tagged wild type (**b**), BB-loop mutants (**f**) and AE-interface mutants (**g**) of RPP1-TIR, RBA1 and TX14. Ten leaves from five plants were detected for each infiltration. Scale bars, 50 μm. The experiments were repeated at least three times with similar results. **c**–**e**, Images and quantification of RPP1-TIR–GFP (**c**), RBA1–GFP (**d**) and TX14–GFP (**e**) FRAP. The photobleaching pulse ends at 0 s. Scale bars, 5 μm. Fluorescence intensities are plotted relative to the pre-bleach time point (*t* = −2 s). Data are mean ± s.d. (*n* = 3 biologically independent samples). **h**, Relative NADase activities of the RPP1 resistosome. The NADase activity of the wild-type RPP1 resistosome was normalized to 100%. Data are mean ± s.d. (*n* = 3 biologically independent samples). **k**, Confocal images of *N. benthamiana* transiently expressing RBA1–GFP, RBA1(BB-loop)–GFP, RBA1(E86A)–mCherry and co-expressing RBA1(BB-loop)–GFP and RBA1(E86A)–mCherry, respectively. Scale bar, 50 μm. Images show a projection of fluorescent images acquired along the *z*-axis. Source Data

**Extended Data Fig. 3:**
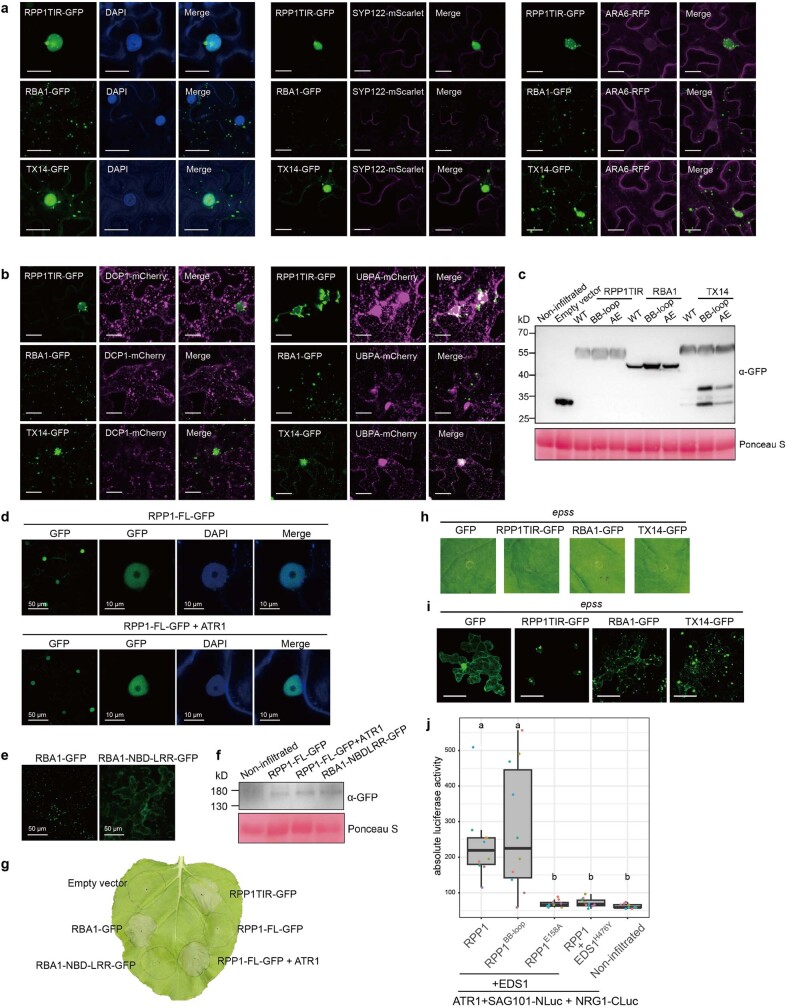
The subcellular localization of TIR condensates and TIR condensation in *epss N. benthamiana*. **a**, Left, confocal images of DAPI-stained *N. benthamiana* transiently expressing RPP1-TIR-GFP, RBA1-GFP and TX14-GFP. Middle, Confocal images of *N. benthamiana* transiently co-expressing GFP-tagged TIRs and the plasma membrane marker SYP122-mScarlet. Right, Confocal images of *N. benthamiana* transiently co-expressing GFP-tagged TIRs and the endomembrane marker ARA6-RFP. Scale bar = 50 μm. Images on the left and right show a projection of fluorescent images acquired along the z-axis. **b**, Confocal images of *N. benthamiana* transiently co-expressing GFP-tagged TIRs and P-body marker DCP1-mCherry (left panel) or stress granule marker UBPA-mCherry after heat shock at 42 °C for 5 min (right panel). Scale bar = 20 μm. Images show a projection of fluorescent images acquired along the z-axis. **c**, Western blot analysis of total N. *benthamiana* leaf protein extracts at 2 dai probed with anti-GFP antibodies. Ponceau S indicates equal loading of total leaf proteins on the blot. **d**, Confocal images of *N. benthamiana* transiently expressing full-length RPP1 without or with the cognate effector ATR1. Left lane: views under 40× objective. Scale bar = 50 μm. Images show a projection of fluorescent images acquired along the z-axis. Right three lanes: enlarged views of nucleus shows colocalization of RPP1-FL-GFP and DAPI-stained nucleus. Scale bar = 10 μm. **e**, Confocal images of *N. benthamiana* transiently expressing RBA1-GFP and RBA1-NBD-LRR-GFP. Scale bar = 50 μm. Images show a projection of fluorescent images acquired along the z-axis. **f**, Western blot analysis of total *N. benthamiana* leaf protein extracts at 2 dai probed with anti-GFP antibodies. Ponceau S indicates equal loading of total leaf proteins on the blot. **g**, Cell death phenotype of *N. benthamiana* leaves transiently expressing TIR-NBD-LRR fusions. Photograph shows a representative leaf after agro-infiltration at 4 dai. **h**, Photographs of representative leaf zones of *epss N. benthamiana* plants transiently expressing GFP-tagged RPP1-TIR, RBA1 and TX14 at 4 dai. **i**, Confocal images of *epss N. benthamiana* transiently expressing GFP-tagged RPP1-TIR, RBA1 and TX14 at 2 dai. At least five leaves were detected for each infiltration, all showing the similar results. Scale bar = 50 μm. Images show a projection of fluorescent images acquired along the z-axis. **j**, Split-luciferase assay of TIR-induced EDS1-SAG101 interaction with NRG1. The Split-Luc N and Split-Luc C were fused to SAG101 and NRG1, respectively. RPP1 (wild type or different variants), ATR1, EDS1 (wild type or small molecule binding site mutant), SAG101 and NRG1 were co-expressed in *epss N. benthamiana*. Results from five independent experiments are displayed (n = 10 biologically independent samples; analyzed by one-way ANOVA with Tukey’s HSD test, *p* = 0.05). For the box plots, the centre line indicates the median, the bounds of the box show the 25th and the 75th percentiles, and the whiskers indicate 1.5 × IQR. The experiments in **a**–**i** were repeated at least three times with similar results. Source Data

We next investigated whether TIR domain proteins form phase condensates in vivo. We first transiently expressed GFP-tagged RPP1-TIR, RBA1 and TX14 in *N. benthamiana*. Consistent with previous data^10,22,23^, expression of these three TIR domain proteins resulted in a cell death phenotype in *N. benthamiana* leaves, albeit with varying activities as determined by ion-leakage assays (Fig. 3a). Imaging of leaf cells by confocal microscopy showed that RPP1-TIR formed nuclear and perinuclear puncta (Fig. 3b). In further support of our in vitro data (Fig. 1c), FRAP assays showed that RPP1-TIR–GFP puncta displayed rapid fluorescence recovery within seconds (Fig. 3c,d), indicating that RPP1-TIR is dynamic, a hallmark feature of liquid–liquid phase separation^28^ (LLPS). In contrast to RPP1-TIR–GFP, GFP-tagged RBA1 and TX14 formed cytoplasmic and nuclear-cytoplasmic puncta, respectively (Fig. 3b and Extended Data Fig. 3a). Cytoplasmic punctate bodies of RBA1 have also been observed in a previous study^23^. Similarly, *Arabidopsis* TX21 formed cytoplasmic puncta when expressed in *N*. *benthamiana*^20^. FRAP experiments showed that RBA1 and TX14 exhibited lower dynamics than RPP1-TIR (Fig. 3d,e), which is consistent with the in vitro results (Extended Data Fig. 1j). The TIR condensates did not co-localize with any of the tested plasma membrane, endomembrane, stress granule or processing-body (P-body) markers (Extended Data Fig. 3a,b), suggesting that the observed TIR condensates are autonomous structures lacking known membranes. Together, these results indicate that TIR domain proteins form condensates in vivo.

To test whether condensation is required for TIR-triggered cell death, we expressed RPP1-TIR(BB-loop), RBA1(BB-loop) and TX14(BB-loop) in *N. benthamiana*. Confocal microscopy analyses of leaf cells of these plants showed that the nuclear condensates of RPP1-TIR mutants were reduced in size and number, and the proteins were more evenly distributed in the cytoplasm and nucleus (Fig. 3f). Notably, the RBA1(BB-loop) and TX14(BB-loop) mutants were expressed homogenously without forming detectable condensates (Fig. 3f and Extended Data Fig. 3c). These results suggest that the BB-loop is critical for TIR condensate formation in plants. The cell death-inducing activity of the BB-loop mutants was also abolished (Fig. 3a), supporting an essential role of phase separation facilitated by the BB-loop in TIR domain-triggered cell death in *N. benthamiana*. Plant TIR BB-loop mutations in RBA1, RPS4-TIR and *Brachypodium distachyon* TIR have been reported to result in a loss of *EDS1*-dependent cell death in *N. benthamiana*^39^. In further support of this conclusion, the loss-of-function mutations RPP1-TIR(AE), RBA1(AE) and TX14(AE) strongly reduced formation of phase condensates in *N. benthamiana* (Fig. 3a,g and Extended Data Fig. 3c). Furthermore, fusion of the nucleotide-binding domian (NBD) and leucine-rich repeat (LRR) segment of RPP1 to the C-terminal end of RBA1 disrupted RBA1 condensation and compromised *RBA1*-trigerred cell death in *N. benthamiana* (Extended Data Fig. 3d–g). Together, these results show that condensate formation is essential for TIR domain protein-mediated cell death in plants.

We tested RPP1-TIR–GFP, RBA1–GFP and TX14–GFP expression in a *N. benthamiana* mutant lacking *EDS1* and its paralogues *PAD4 and SAG101a* and *SAG101b*^40^ (*eds1 pad4 sag101a sag101b* (*epss*)). As anticipated, the cell death activity of the three TIR domain proteins was abolished in *epss N. benthamiana* (Extended Data Fig. 3h). However, phase separation of these TIR domain proteins in *epss* plants was similar to that in wild-type *N. benthamiana* (Extended Data Fig. 3i). These data show that phase condensation is upstream of *EDS1*-dependent cell death activity of TIRs and that TIR condensates per se do not induce cell death.

The above data do not exclude the possibility that the greatly compromised NADase and cell death activities of the BB-loop mutants of TIR domain proteins (Figs. 2d and 3a) result from impaired active sites. This model, however, is argued against by the observation that the equivalent BB-loop mutations of full-length RPP1 (E122A/R123A/S124A/K125A/S126A (RPP1(BB-loop))) only slightly reduced its NADase activity (Fig. 3h). Consistently, split-luciferase assays further showed that RPP1(BB-loop) was still able to induce EDS1–SAG101 interaction with NRG1 (Extended Data Fig. 3j). Moreover, RPP1(BB-loop) retained wild-type-like cell death activity when co-infiltrated with its recognized pathogen effector ATR1 in *N. benthamiana*^2^ (Fig. 3i). One plausible explanation for the distinct effects of BB-loop mutations on NADase activities of the RPP1 resistosome and RPP1-TIR is that full-length RPP1 mutants retain an intact nucleotide-oligomerization domain that is sufficient for formation of composite catalytic sites, whereas the same mutations can disrupt the oligomerization and phase separation of TIR domain proteins that is required for their enzymatic activity.

**Extended Data Fig. 4:**
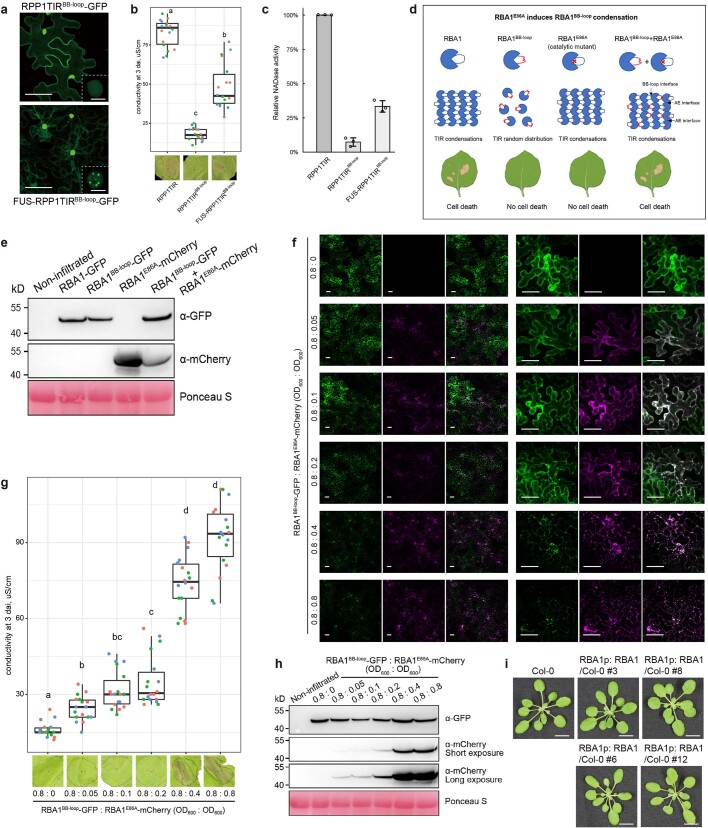
Rescuing TIR^BB-loop^-triggered cell death by FUS and condensation-active TIR proteins in *N. benthamiana*. **a**, Confocal images of *N. benthamiana* transiently expressing GFP-tagged RPP1-TIR^BB-loop^ and FUS-RPP1TIR^BB-loop^. Scale bar = 50 μm. The nucleus was shown in the corner. Scale bar = 10 μm. Images show a projection of fluorescent images acquired along the z-axis. **b**, Cell death phenotype of *N. benthamiana* plants transiently expressing GFP-tagged RPP1TIR^BB-loop^ and FUS-RPP1TIR^BB-loop^. Top: Ion leakage assay at 3 dai. Results from three independent experiments are shown (n = 18 biologically independent samples; analyzed by one-way ANOVA with Tukey’s HSD test, *p* = 0.001). Bottom: Photograph shows a representative leaf after agro-infiltration at 4 dai. **c**, The NADase activity of RPP1TIR^BB-loop^ protein and FUS- RPP1-TIR^BB-loop^ protein. The NADase activity of the wild type RPP1-TIR was normalized to 100%. Data are mean ± SD (n = 3 biologically independent samples). **d**, The schematic for the rationale of RBA1^E86A^-induced RBA1^BB-loop^ condensation. The cartoon images were created with BioRender.com. **e**, Western blot analysis of total *N. benthamiana* leaf protein extracts at 2 dai probed with anti-GFP and anti-mCherry antibodies. Ponceau S indicates equal loading of total leaf proteins on the blot. **f**, Confocal images of *N. benthamiana* leaves infiltrated with GFP-tagged RBA1^BB-loop^ and mCherry-tagged RBA1^E86A^ at different ratios. The numbers indicate the concentration (OD_600_) of each *Agrobacterium* solution used for infiltration. Left: views under 10× objective. Scale bar = 50 μm. Right: views under 40× objective. Scale bar = 50 μm. Images show a projection of fluorescent images acquired along the z-axis. **g**, Cell death phenotype of *N. benthamiana* plants infiltrated with GFP-tagged RBA1^BB-loop^ and mCherry-tagged RBA1^E86A^ at different ratios. Top: Ion leakage assay at 3 dai. Results from three independent experiments are shown (n = 18 biologically independent samples; analyzed by one-way ANOVA with Tukey’s HSD test, *p* = 0.001). Bottom: Photograph shows a representative leaf after agro-infiltration at 4 dai. **h**, Western blot analysis of total *N. benthamiana* leaf protein extracts at 2 dai probed with anti-GFP and anti-mCherry antibodies, respectively. The long exposure panel of anti-mCherry displays the low expressions of mCherry-tagged RBA1^E86A^ in 0.8:0.05 and 0.8:0.1 samples, which are invisible on the short exposure panel. Ponceau S indicates equal loading of total leaf proteins on the blot. **i**, Images of 3-week-old transgenic *Arabidopsis* plants *RBA1p*:RBA1/Col-0 and RBA1p:RBA1^BB-loop^/Col-0. Scale bar = 1 cm. For the box plots, the centre line indicates the median, the bounds of the box show the 25th and the 75th percentiles, and the whiskers indicate 1.5 × IQR. The experiments in **a**, **e**, **f** and **h** were repeated at least three times with similar results. Source Data

The data presented above suggest that the BB-loop mutations affect the condensation of TIR domain proteins, whereas their catalytic activity remains unaffected. If this model holds true, restoring the phase separation of these BB-loop mutant proteins would rescue their enzymatic and cell death activities. To test this prediction, we fused the N-terminal IDR (residues 1–212) of the human RNA-binding protein FUS, which has well-established LLPS activity^41^, to RPP1-TIR(BB-loop). The FUS fusion substantially enhanced the LLPS activity of RPP1-TIR(BB-loop) in *N. benthamiana* (Extended Data Fig. 4a). Accordingly, the FUS-fused RPP1-TIR(BB-loop) displayed much higher NADase and cell death activities than RPP1-TIR(BB-loop), albeit still lower than wild-type RPP1-TIR (Extended Data Fig. 4b,c). The catalytic mutant RBA1(E86A) possesses an intact BB-loop, whereas the RBA1(BB-loop) mutant retains an intact catalytic site. Interaction of the two RBA1 mutants can lead to the formation of a composite RBA1 catalytic centre, resulting in cell death (Extended Data Fig. 4d). As anticipated, individual expression of mCherry-fused RBA1(E86A) or GFP-fused RBA1(BB-loop) did not induce cell death (Fig. 3j) and RBA1(E86A), but not RBA1(BB-loop), formed condensates (Fig. 3k) in *N. benthamiana*. By contrast, co-expression with RBA1(E86A) significantly induced condensation of RBA1(BB-loop) and, more importantly, fully rescued the cell death activity of RBA1(BB-loop) in *N. benthamiana* (Fig. 3j,k and Extended Data Fig. 4e). Similar to wild-type RBA1, the condensation and cell death in these co-expression experiments were protein concentration-dependent (Extended Data Fig. 4f–h). Together, these results support the conclusion that the BB-loop mutations of TIR proteins specifically disrupt phase separation, subsequently compromising the enzymatic and cell death activities of TIR domain proteins.

## TIR condensates and cell death in planta

**Fig. 4:**
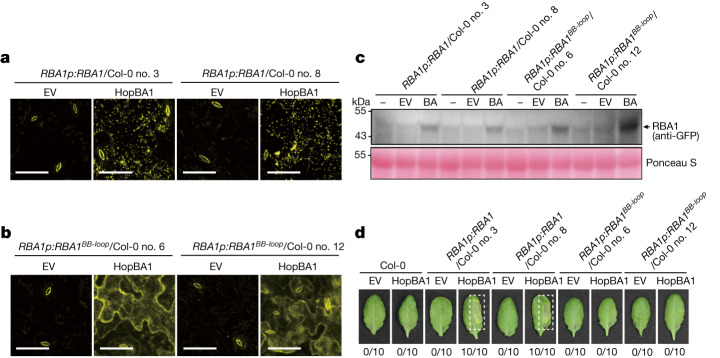
Pathogen-induced RBA1 condensates mediate cell death in *Arabidopsis*. **a**,**b**, Confocal images of *RBA1p*:*RBA1*/Col-0 transgenic lines (no. 3 and no. 8) (**a**) and *RBA1p*:*RBA1*^*BB-loop*^/Col-0 transgenic lines (no. 6 and no. 12) (**b**) *Arabidopsis* plants 24 h after inoculation with Pf0-1 with empty vector (EV) or expressing HopBA1. The fluorescence intensity of the images was calibrated by the autofluorescence in the guard cells. Ten leaves from five plants were detected for each treatment, showing similar results. Scale bars, 40 μm. **c**, Western blot analysis of total leaf protein extracts of transgenic *RBA1p*:*RBA1*/Col-0 and *RBA1p*:*RBA1*^*BB-loop*^/Col-0 *Arabidopsis* plants 24 h after inoculation. RBA1–GFP and RBA1(BB-loop)–GFP were probed with GFP antibody. Ponceau staining was used to indicate equal loading of samples. BA, inoculation with Pf0-1 expressing HopBA1; EV, inoculation with Pf0-1 expressing empty vector; −, no inoculation. Experiments were repeated at least three times with similar results. **d**, Visual signs of cell death in leaves of transgenic *RBA1p*:*RBA1*/Col-0 and *RBA1p*:*RBA1*^*BB-loop*^/Col-0 *Arabidopsis* plants 36 h after inoculation with Pf0-1 expressing empty vector (EV) or *HopBA1*. The numbers on the bottom indicate the proportion of leaves exhibiting cell death out of all inoculated leaves.

Our results show that condensation is critical for the cell death activity of TIR domain proteins, when over-expressed in *N. benthamiana*. To further assess the physiological relevance of phase condensation in pathogen effector-triggered and TIR domain-mediated cell death in planta, we first generated wild-type mYFP-fused *RBA1* or *RBA1*^*BB-loop*^ transgenic lines in *Arabidopsis* accession Col-0 driven by native 5′ regulatory sequences^23^ (1,733 bp of genomic DNA sequence of accession Ag-0 harbouring *RBA1*), which we designated *RBA1p:RBA1*/Col-0 and *RBA1p:RBA1*^*BB-loop*^/Col-0. Similar to wild-type Col-0, non-inoculated transgenic *RBA1* plants exhibited no cell death phenotype (Extended Data Fig. 4i). We then inoculated the *RBA1* transgenic plants with *Pseudomonas fluorescens* strain Pf0-1 expressing the effector HopBA1 or an empty vector. Delivery of HopBA1 by Pf0-1 in Col-0 leaves, but not Pf0-1 containing empty vector, strongly induced RBA1 expression (Fig. 4a,c), supporting previous results showing that the effector is needed to stimulate *RBA1* expression via native promoter sequences^23^. Notably, RBA1 formed condensates in the Pf0-1-HopBA1-infected leaves, as determined by confocal microscopy (Fig. 4a). This result is consistent with data from *N. benthamiana* in which RBA1 was transiently expressed by a strong promoter (Fig. 3b). Collectively, these data led us to conclude that the RBA1 TIR has phase-separation activity that can be induced by the pathogen effector HopBA1 in *Arabidopsis*.

Similar to the *RBA1p:RBA1*/Col-0 line, the *RBA1p:RBA1*^*BB-loop*^/Col-0 transgenic *Arabidopsis* plants exhibited only marginal expression of the RBA1(BB-loop) mutant protein following treatment with empty vector, but RBA1(BB-loop) expression was strongly induced in response to HopBA1, with levels similar to those in RBA1(WT) plants (Fig. 4b,c). In contrast to the *RBA1p:RBA1*/Col-0 lines, no condensates were detectable in the *RBA1p:RBA1*^*BB-loop*^/Col-0 transgenic plants, even though expression of the mutant protein was similar to that in RBA1(WT) plants (Fig. 4b,c). Instead, the RBA1(BB-loop) mutant protein appeared to be evenly distributed throughout the cytoplasm. These results establish a critical role for the BB-loop in mediating RBA1 phase condensation during pathogen infection. We next evaluated HopBA1-trigged cell death in the *RBA1* transgenic lines. At 36 h post inoculation (hpi) with Pf0-1 HopBA1, *RBA1p:RBA1*/Col-0 plants, but not *RBA1p:RBA1*^*BB-loop*^/Col-0 plants, showed a strong cell death response in Pf0-1 inoculated leaves (Fig. 4d). These results suggest that HopBA1-induced RBA1 phase condensates are essential for the cell death activity of the TIR-only protein in *Arabidopsis*.

## Discussion

Mounting evidence supports a critical role for truncated TIR proteins in plant immunity. However, the mechanism underlying TIR activation remains unknown owing to the lack of ligand-sensing domains and/or the absence of strain-specific pathogen effectors. In the current study, we provide in vitro and in planta evidence that substrate-induced phase separation promotes TIR NADase activity and activates TIR immune responses in plants. This model does not contradict the hypothesis that TIR-only proteins can act as adapters in immunity signalling^19^. Several adapter proteins, such as ASC, have been shown to have filament activity in amplifying immune signalling in animals^42^. Phase separation is also critical for cell survival during plant immunity^43^ and temperature-sensitive plant immunity^44,45^, suggesting a broad role of phase separation in regulating plant immune responses. Notably, the formation of condensates is a recurring theme in enabling signal transduction and amplification in animal immunity^29,46^.

**Extended Data Fig. 5:**
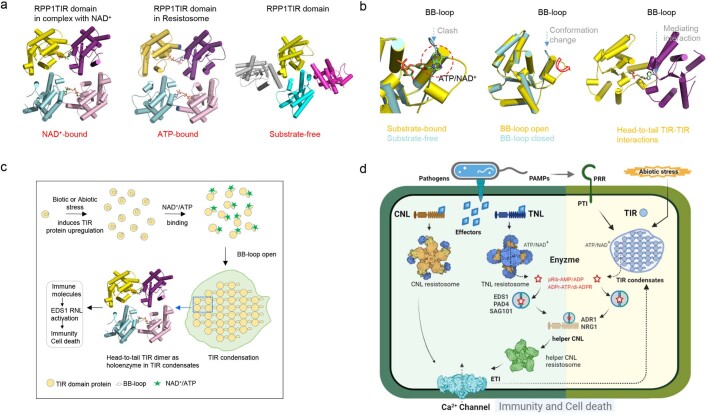
Mechanism of substrate-induced condensation of TIR domain proteins. **a**, Tetrameric structure of the RPP1-TIR domain. Shown in the left, middle and right panels are the crystal structure of the RPP-TIR domain in a substrate-free form (PDB 5TEB), the crystal structure of the RPP1-TIR domain in an NAD^+^-bound form (PDB 7XOZ) and Cryo-EM structure of the RPP1-TIR domain from the RPP1 resistosome (PDB 7CRC). **b**, Substrate binding induces BB-loop open to mediate TIR-TIR interaction. The closed BB-loop blocks substrate-binding site (left panel). Substrate binding triggers BB-loop open (middle panel). Opened BB-loop mediates TIR-TIR interactions (right panel). **c**, A proposed mechanism for substrate-induced condensation to activate TIR domain signaling. Biotic or abiotic stresses induce TIR domain protein upregulation. Binding of ATP/NAD^+^ induces conformational change of BB-loop and phase separation of TIR domain proteins. The TIR condensation stabilizes head-to-tail TIR dimers as catalytically active centers to catalyze ATP/NAD^+^ hydrolysis, producing small molecules for TIR immune signaling. **d**, A schematic of working model on phase separation-activated TIR-only signaling linking PTI, ETI and abiotic stress response. The cartoon images were created with BioRender.com.

Phase separation can enhance enzyme activity, typically by concentrating and organizing enzyme assemblies^26,27,47^. The crystal structure of the RPP1-TIR domain in complex with NAD^+^ showed that NAD^+^ binding induces a conformational change in the BB-loop, from a closed state to an open state^15^. The open BB-loop mediates the formation of two head-to-tail TIR dimers, as found in the cryo-electron microscopy structure of the RPP1 resistosome (Extended Data Fig. 5a,b). Combined with our biochemical data (Fig. 2c), we propose that substrate binding-induced conformational change in BB-loops mediates high-order TIR–TIR interactions, triggering TIR condensation, in which the head-to-tail TIR dimers function as holoenzymes for catalysing NAD^+^ hydrolysis (Extended Data Fig. 5c). This mechanism is reminiscent of the activation of cGAMP activity of cGAS by its double stranded DNA (dsDNA) substrate in animals^29^. TIR domain proteins also catalyse the production of 2′,3′-cNMPs with double stranded RNA or dsDNA as substrates in vitro^48^. Binding of the RNA or DNA-type substrates results in the formation of TIR filament structures to induce TIR 2′,3′-cNMP synthetase activity^48^. Notably, phase separation is also important for NADase activity of the human SARM1 TIR and the *C. elegans* SARM1/TIR-1^49^, whereas filament activity is required for activation of NADase activity of bacterial TIR proteins^50–52^. Thus, phase separation- or filament activity-mediated higher-order assembly are likely to be evolutionarily conserved mechanisms for TIR proteins to modulate their enzymatic activities.

In addition to multi-valency and IDRs, phase separation of macromolecules also strongly depends on their concentrations^53^. Given the essential role of phase separation in TIR-mediated cell death, this suggests that increased concentrations of a TIR domain protein can promote its activation, as supported by biochemical and in planta data (Extended Data Figs. 1f and 4f–h). Induction of phase separation and activation of the TIR-only protein by HopBA1-upregulated *RBA1* expression is consistent with the observation that cell death activity of RBA1 is tightly correlated with its protein level^23^. In this model, specific recognition of the *P. syringae* effector HopBA1 in *A. thaliana* accession Ag-0 is the consequence of inter-accession variation in the responsiveness of *RBA1* expression to HopBA1 delivery into plant cells. In this context, the cell death activity of the *A. thaliana* TNL RPS4 is also strictly dependent on its protein level in plants^54^. Moreover, in an activation-tagging system, a DNA tag in the promoter region of *A. thaliana TX12* enhances expression of this *TIR*-only gene and activates *EDS1*-dependent immune responses^55^, suggesting that upregulation of TIR protein expression is sufficient for their activation. A similar activation mechanism might be used by TIRs that are transcriptionally induced during PTI^10^. Indeed, one of the PTI-induced TIRs, TX14, has phase-separation activity required for its cell death phenotype when expressed in *N. benthamiana* (Fig. 3b). Activation of TIRs induced by PTI elicitors in turn boosts PTI signalling in an *EDS1*-dependent manner, which can be a mechanism for PTI–ETI cross-potentiation^8,9^. In addition, EDS1–PAD4 dimers and ADR1s contribute to the disease-resistance activity of some CNLs, suggesting a role of TIR signalling in CNL-triggered defence potentiation^56^. Thus, TIR domain protein signalling activated by phase separation may contribute to establishing crosstalk between immune signalling branches (Extended Data Fig. 5d). This in turn could make it more difficult for pathogens to disable immune signalling, resulting in increased robustness of the plant immune system.

## Methods

### Plant materials and growth conditions

*A. thaliana* accession Col-0 and *N. benthamiana* were used in this work. The *N. benthamiana* quadruple mutant *epss* (*eds1 pad4 sag101a sag101b*) was described previously^40^. The RBA1 transgenic *Arabidopsis* was generated in this study. *Arabidopsis* plants were grown in an incubator with a 10 h:14 h light:dark cycle at 22 °C in 70% humidity for 4–5 weeks. *N. benthamiana* plants were grown in a greenhouse under long-day conditions for 4–5 weeks.

### Plasmid constructs and transient expression in *N. benthamiana*

For *N. benthamiana* transient expression, the TIR domain of RPP1 (residues 1–248), RBA1 (residues 1–191), TX14 (residues 1–220) and FUS protein fused RPP1-TIR(BB-loop) were cloned into the pCHF3-GFP vector and RBA1(E86A) was cloned into pAMPAT-mCherry. Site-directed mutagenesis of RPP1-TIR(BB-loop), RPP1-TIR(AE), RBA1(BB-loop), RBA1(AE), RBA1(E86A), TX14(BB-loop) and TX14(AE) was generated using KOD-Plus-Mutagenesis Kit (TOYOBO).

*Agrobacterium* strains (GV3101) harbouring the constructs of interests were cultured in liquid Luria-Bertani medium overnight. The dense cultures were inoculated into fresh medium by 1:100 dilution and incubated for 6–8 h. The bacteria were sedimented and resuspended in infiltration buffer (10 mM MgCl_2_, 10 mM MES-KOH, pH 5.7) to an OD_600_ of 0.2–0.8. The resuspended agrobacteria were infiltrated into tobacco leaves using 1-ml syringes without needles.

### Cell death quantification in *N. benthamiana*

For measuring conductivity^57^, six 8 mm leaf discs from *N. benthamiana* agroinfiltrated leaves were taken at 3 dpi, washed in 10 ml of milliQ water (18.2MV*cm, mQ) for 30 min, transferred to a 24-well plate with 1 ml milliQ water in each well, and incubated at room temperature. Electrolyte leakage was measured at 0 and 6 h with a conductometer Horiba Twin Model B-173. Statistical analysis was performed on conductivity data via Tukey’s HSD test. For visual assessment of cell death symptoms, images of agrobacteria-infiltrated leaf spots were taken at 4–5 dpi.

### Plasmid constructs and *Arabidopsis* transformation

To construct *RBA1p:RBA1*, the native *RBA1* promoter (800 bp)^23^ of accession Ag-0 fused with RBA1 cDNA (1–573 bp) was synthesized, and then cloned into the pENTR/D-TOPO vector (Thermo Fisher Scientific, K2400200). The mutants of *RBA1pro:RBA1*^*BB-loop*^ were generated using KOD-Plus-Mutagenesis Kit (TOYOBO). The obtained plasmids were LR-recombined into the pXCG vector with a C-terminal mYFP tag. Transgenic seedlings were obtained through *Agrobacterium*-mediated transformation of *A. thaliana* using the floral dip method in wild-type Col-0. Transgenic plants were screened with Basta. Homozygous lines were identified by calculating offspring segregation. Two independent lines for each transgenic material were used in the experiments.

### HopBA1 delivery and cell death assays in *Arabidopsis*

Pf0-1 bacteria carrying HopBA1 or empty vector were grown at 28 °C overnight. The bacteria were resuspended in 10 mM MgCl_2_ to OD_600_ of 0.2 and infiltrated with a needleless syringe into rosette leaves of 5- to 6-week-old *RBA1* transgenic *Arabidopsis* plants. For visual assessment of cell death symptoms, images of infiltrated leaf spots were taken at 36 hpi.

### Western blot analysis

The *Arabidopsis* leaves were rapidly frozen in liquid nitrogen after collection, and ground into fine powder. A fraction of powder was added with an equal volume of 2× SDS sample buffer (4% SDS, 100 mM Bis-Tris pH 6.8, 10% glycerol, 2% β-mercaptoethanol), and incubated on ice for 15 min, following by boiling at 75 °C for 10 min. After centrifugation at 13,000*g* for 5 min, the protein extracts were fractionated by 10% SDS–PAGE, blotted onto a PVDF membrane. Immunoblot assay was then performed using a anti-GFP (Takara Clontech, 632381, 1:5,000), anti-mCherry (Agrisera, AS184179, 1:2,000) and polyclonal goat anti-mouse IgG–HRP (Santa Cruz Biotechnology, sc2005, 1:5,000).

### Recombinant TIR protein expression and purification

GST-tagged RPP-TIR (residues 60–254) was cloned into the pFastBac1 (Invitrogen) with an N-terminal GST tag^2^. The construct was used for generating recombinant baculovirus in sf21 insect cells (Invitrogen). RPP1-TIR was expressed in sf21 insect cells with recombinant baculovirus infection at 28 °C for 60 h. The infected cells were collected and lysed by sonification in buffer (25 mM Tris-HCl, 150 mM NaCl, pH 8.0). The cell lysates were centrifuged at 30,000*g* for 90 min. The supernatant containing soluble proteins were collected and allowed to flow through Glutathione Sepharose 4B resin (GE Healthcare). After washing with 2 column volumes of sonification buffer, the fusion proteins were incubated with PreScission protease at 4 °C overnight to remove the N-terminal GST tag. The digested RPP1-TIR proteins flowed through the columns in the buffer (25 mM Tris-HCl, 150 mM NaCl, pH 8.0).

GFP-tagged RPP-TIR (residues 1–254), RBA1 (residues 1–191), TX14 (residues 1–351) (wild-type, BB-loop mutant, and AE interface mutants) and FUS-RPP1-TIR^BB-loop^ were cloned into the pET-MBP-mGFP vector. All the constructs were transformed into *E. coli* BL21 (DE3) competent cells with 90 s heat shock at 42 °C. The *E. coli* cells were cultured in LB liquid medium at 37 °C to OD_600_ of 0.8. 0.5 mM isopropyl β-d-1-thiogalactopyranoside was added to induce protein expression at 16 °C for 16 h. The cells were collected by centrifugation and lysed by sonication in buffer (25 mM Tris-HCl, 150 mM NaCl, pH 8.0). The cell lysates were centrifuged at 30,000*g* for 90 min. The supernatants were initially purified by Ni-NTA (GE healthcare) affinity beads, and subsequently purified on a Superdex 200 Increase 10/300 column (SD200). The proteins were eluted in the buffer (25 mM Tris-HCl, 100 mM NaCl, pH 8.0). A centrifugal filter (Amicon ultra) was used for protein concentration and buffer exchange.

### In vitro phase-separation assay

In vitro phase-separation assays were performed in buffer containing 25 mM Tris-HCl (pH 8.0) and 100 mM NaCl. Other buffer conditions were indicated in the figure legends. All protein samples were centrifuged at 12,000*g* to remove the potentially denatured protein pellets in the bottom before performing the phase-separation assay. Protein concentrations were determined by NanoDrop spectrophotometry (IMPLEN NP80). Protein phase separation reacted in 1.5-ml Eppendorf tubes. Liquid droplets were observed using confocal microscope Zeiss LSM 880 equipped with ×20 and ×40 objectives.

### NADase assay

Purified wild-type RPP1-TIR, RBA1 and TX14 and mutant proteins (their concentrations are indicated in the figure legends) were used for NADase assays. Proteins were individually incubated with 100 μM NAD^+^ and 10 mM MgCl_2_ in the buffer containing 100 mM NaCl, 25 mM Tris-HCl pH 8.0 at 25 °C for 16 h. After reaction, samples were centrifuged and immediately applied for high-performance liquid chromatography (HPLC) analysis^16^. HPLC was performed on an Agilent 1260 bioinert HPLC system using a Synergi Fusion-RP 80 A° (4.6 3 150 mm, 4 mm) (Phenomenex) column. The samples were measured via an 8-min method. Samples (10 μl) were injected at 550 ml min^−1^ with ammonium formate (5 mM) in water and methanol used as mobile phases A and B, respectively. The elution profile was as follows: 0 to 3 min, 10 to 70% B; 3 to 6 min, 70% B; 6 to 6.1 min, 70 to 10% B; 6.1 to 8 min, 10% B. UV signals were detected at 260 nm. Reference standards were used to determine respective retention times. The integrations of peak areas were used to calculate relative concentrations.

### Microscopy imaging

Imaging for *N. benthamiana* and *Arabidopsis* leaves were performed with Zeiss LSM 880 inverted confocal laser scanning microscope using a 40× water objective. GFP was detected using 488 nm laser excitation and 500–540 nm emission filter. YFP was detected using 514 nm laser excitation and 520–570 nm emission filter. RFP, mCherry and mScarlet were detected using 561 nm laser excitation and 580–660 nm emission filter. The images were collected using *z*-stack mode within a stepwise of 0.2–0.3 μm, and stacked using imageJ 1.53a software. For the DAPI staining, 300 μM DAPI staining solution was infiltrated into *N. benthamiana* leaves 10 min before observation. DAPI was detected using 405 nm laser excitation and 410–500 nm emission filter.

### FRAP

FRAP of TIR condensates in the infiltrated the *N. benthamiana* leaves was performed on a Zeiss LSM 880 confocal microscope using ×40 objective^58^. The punctate region of RPP1-TIR–GFP was bleached using a 488 nm laser pulse. In vitro FRAP analysis was conducted with samples in slices using Zeiss LSM 880 confocal microscope using a ×60 oil objective. Droplets were bleached with a 488 nm laser pulse. The recovery time was recorded for the indicated time as mentioned. Image analysis was made by ZEN 3.2 software. Data were plotted using plotted using Microsoft Excel.

### Luciferase assay

Agrobacteria carrying RPP1 variants, ATR1, EDS1 or EDS1(H476Y)^16^, SAG101–N-terminal luciferase fragment (NLuc) and NRG1–C-terminal luciferase fragment (CLuc) were infiltrated into *N. benthamiana epss* leaves at OD_600_ 0.2. The samples were collected at 2 dai. Three 4-mm leaf disks from 3 independent leaves were pooled per biological replicate and ground to a fine powder. One-hundred microlitres of reporter lysis buffer (+150 mM Tris, pH 7.5) was added to the samples. The samples were mixed in a 1:1 ratio with substrate and luminescence was measured with a luminometer^59^. Data were plotted using R Studio 2021.09.0 software.

### Reporting summary

Further information on research design is available in the Nature Portfolio Reporting Summary linked to this article.

## Online content

Any methods, additional references, Nature Portfolio reporting summaries, source data, extended data, supplementary information, acknowledgements, peer review information; details of author contributions and competing interests; and statements of data and code availability are available at 10.1038/s41586-024-07183-9.

### Supplementary information

Supplementary Figure 1 Uncropped blots and gel images.

Reporting Summary

Supplementary Video 1 ATP instantaneously induces RPP1-TIR protein phase separation.

### Source data

Source Data Figs. 1–3 and Extended Data Figs. 1–4

## Data Availability

All data are available in the main text or the supplementary materials. Uncropped gel and blot source data are provided in Supplementary Fig. 1. Source data are provided with this paper.
