# Supplementary material for: Substrate-induced condensation activates plant TIR domain proteins
Source: Nature. 2024 Mar 13;627(8005):847–53. doi: 10.1038/s41586-024-07183-9 (PMC10972746; doi:10.1038/s41586-024-07183-9)

nature portfolio

https://doi.org/10.1038/s41586-024-07183-9

Supplementary information

Substrate-induced condensation activates plant TIR domain proteins

In the format provided by the authors and unedited

Figure 4c

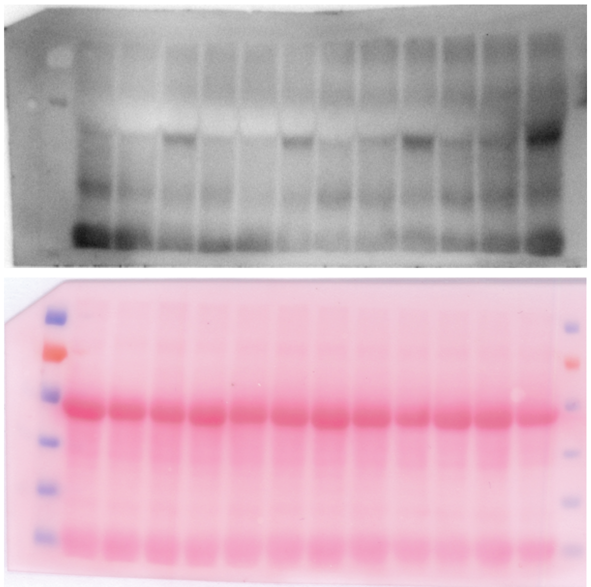

Extended Data Figure 1i

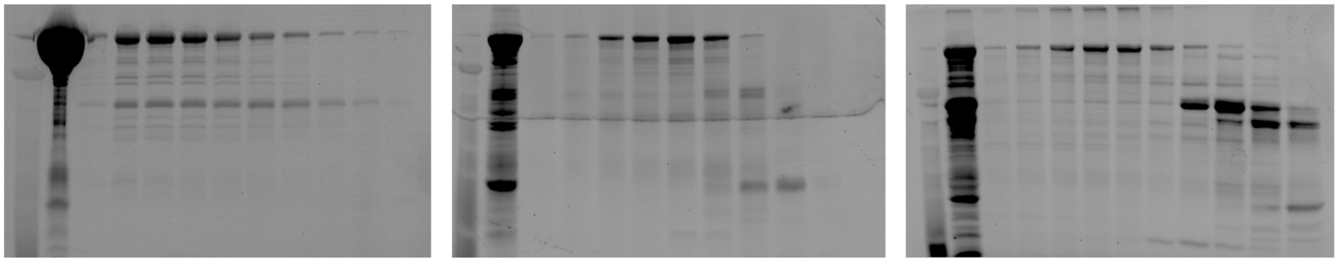

Extended Data Figure 2e

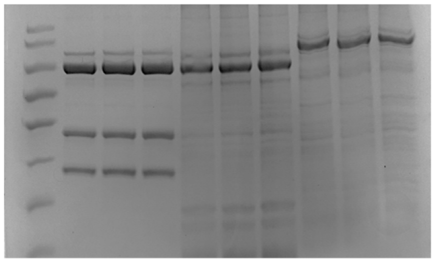

Extended Data Figure 3c

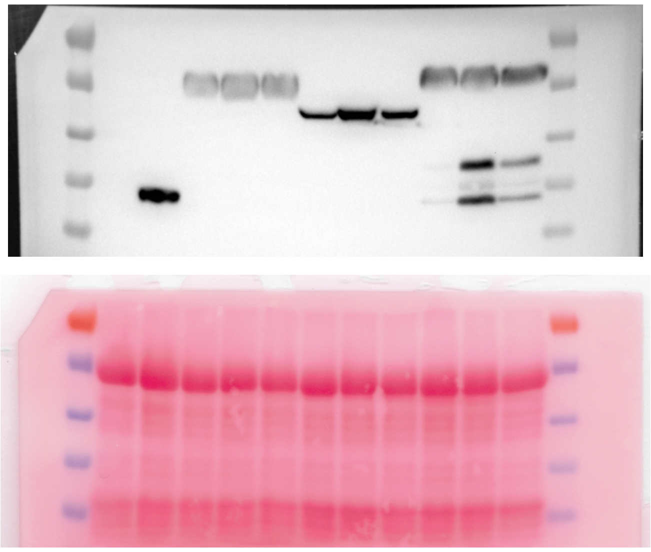

Extended Data Figure 3f

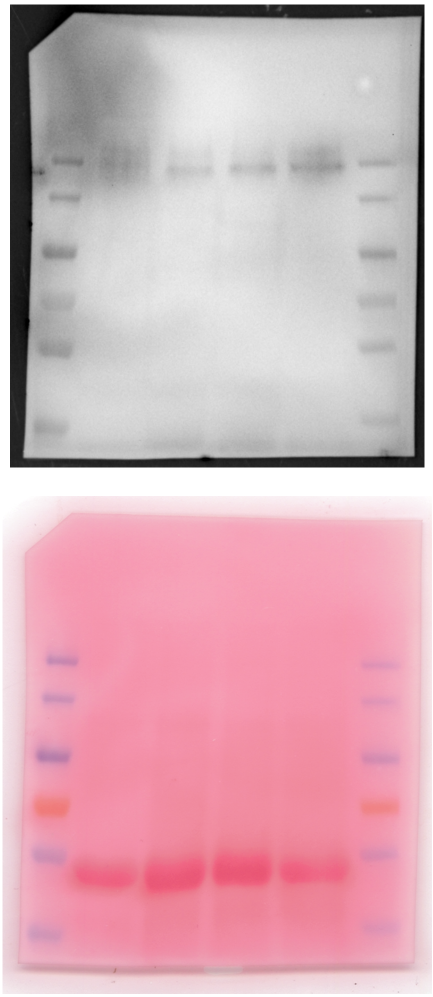

Extended Data Figure 4h

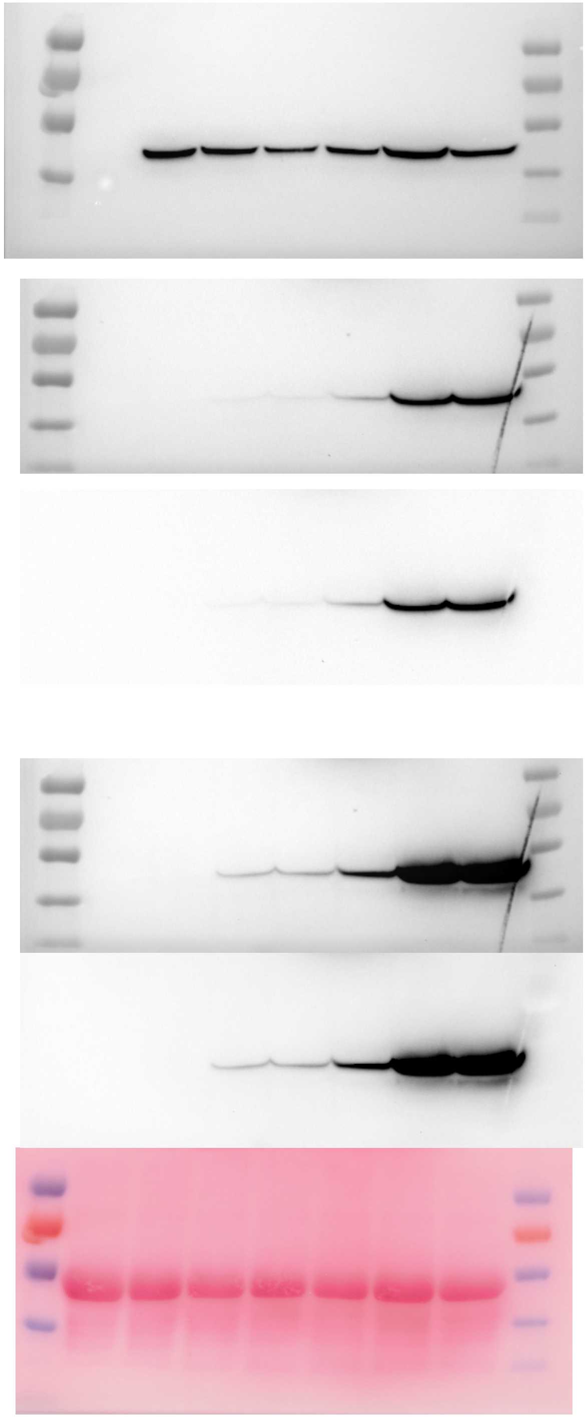

Extended Data Figure 4e

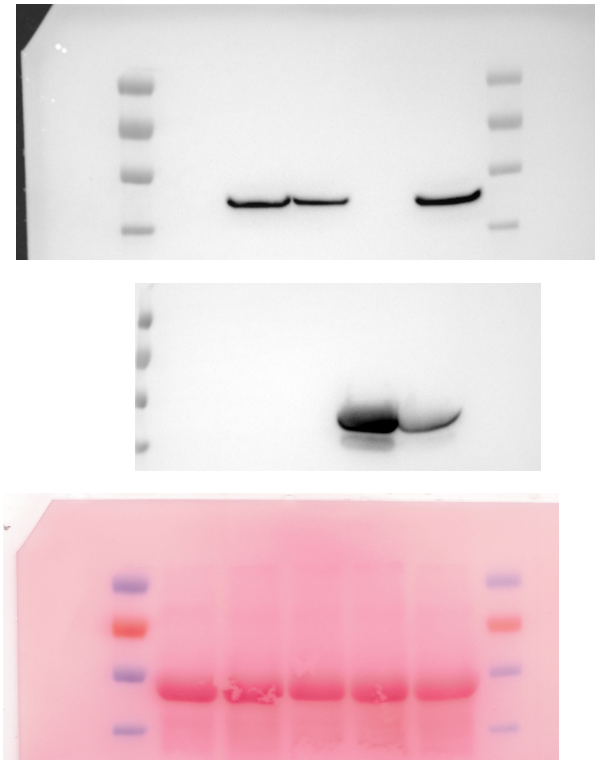

Supplement: Supplementary file 1 — Uncropped blots and gel images. [file 41586_2024_7183_MOESM1_ESM.pdf]
