# Supplementary material for: Substrate-induced condensation activates plant TIR domain proteins
Source: Nature. 2024 Mar 13;627(8005):847–53. doi: 10.1038/s41586-024-07183-9 (PMC10972746; doi:10.1038/s41586-024-07183-9)
Supplement: Supplementary file 2 — Reporting Summary [file 41586_2024_7183_MOESM2_ESM.pdf]

nature portfolio

Corresponding author(s): Jijie Chai
Paul Schulze-Lefert

Last updated by author(s): Jan 11, 2024

Reporting Summary

Nature Portfolio wishes to improve the reproducibility of the work that we publish. This form provides structure for consistency and transparency in reporting. For further information on Nature Portfolio policies, see our Editorial Policies and the Editorial Policy Checklist.

Statistics

For all statistical analyses, confirm that the following items are present in the figure legend, table legend, main text, or Methods section.

n/a	Confirmed	
☐	☒	The exact sample size (*n*) for each experimental group/condition, given as a discrete number and unit of measurement
☐	☒	A statement on whether measurements were taken from distinct samples or whether the same sample was measured repeatedly
☐	☒	The statistical test(s) used AND whether they are one- or two-sided<br>*Only common tests should be described solely by name; describe more complex techniques in the Methods section.*
☒	☐	A description of all covariates tested
☒	☐	A description of any assumptions or corrections, such as tests of normality and adjustment for multiple comparisons
☐	☒	A full description of the statistical parameters including central tendency (e.g. means) or other basic estimates (e.g. regression coefficient) AND variation (e.g. standard deviation) or associated estimates of uncertainty (e.g. confidence intervals)
☐	☒	For null hypothesis testing, the test statistic (e.g. *F*, *t*, *r*) with confidence intervals, effect sizes, degrees of freedom and *P* value noted<br>*Give P values as exact values whenever suitable.*
☒	☐	For Bayesian analysis, information on the choice of priors and Markov chain Monte Carlo settings
☒	☐	For hierarchical and complex designs, identification of the appropriate level for tests and full reporting of outcomes
☒	☐	Estimates of effect sizes (e.g. Cohen's *d*, Pearson's *r*), indicating how they were calculated

Our web collection on statistics for biologists contains articles on many of the points above.

Software and code

Policy information about availability of computer code

Data collection	ZEN, 3.2<br>ImageJ, 1.53a
Data analysis	RStudio, 2021.09.0<br>PyMOL 1.7.2.1,<br>Microsoft Office Software package (Excel) 2016

For manuscripts utilizing custom algorithms or software that are central to the research but not yet described in published literature, software must be made available to editors and reviewers. We strongly encourage code deposition in a community repository (e.g. GitHub). See the Nature Portfolio guidelines for submitting code & software for further information.

Data

Policy information about availability of data

All manuscripts must include a data availability statement. This statement should provide the following information, where applicable:

- Accession codes, unique identifiers, or web links for publicly available datasets
- A description of any restrictions on data availability
- For clinical datasets or third party data, please ensure that the statement adheres to our policy

All data are available in the main text or the supplementary materials. Uncropped gel and blot source data are provided in Supplementary Fig. 1. Source data are provided with this paper.

Research involving human participants, their data, or biological material

Policy information about studies with human participants or human data. See also policy information about sex, gender (identity/presentation), and sexual orientation and race, ethnicity and racism.

Reporting on sex and gender	n/a
Reporting on race, ethnicity, or other socially relevant groupings	n/a
Population characteristics	n/a
Recruitment	n/a
Ethics oversight	n/a

Note that full information on the approval of the study protocol must also be provided in the manuscript.

Field-specific reporting

Please select the one below that is the best fit for your research. If you are not sure, read the appropriate sections before making your selection.

☒ Life sciences ☐ Behavioural & social sciences ☐ Ecological, evolutionary & environmental sciences

For a reference copy of the document with all sections, see nature.com/documents/nr-reporting-summary-flat.pdf

Life sciences study design

All studies must disclose on these points even when the disclosure is negative.

Sample size	The sample size and the results of statistical analyses are described in the relevant figure legends or methods section.<br>1. For conductivity measurement, the sample size was determined based on the previous publication (Ma et al., 2020, Science).<br>2. For visual cell death analysis in N.benthamiana, the sample size was determined based on the previous publications (Ma et al., 2020, Science; Yu et al., 2022, Cell).<br>3. For visual cell death analysis in N.benthamiana, the sample size was determined based on the previous publications (Sun et al., 2021, Nat Commun).<br>4. For Luciferase assay, the sample size was determined based on the previous publications (Joram et al., 2020, MPMI).<br>5. For NADase assay, the sample size was determined based on the previous publications (Ma et al., 2020, Science; Yu et al., 2022, Cell).<br>6. For microscopy imaging and FRAP assay, the sample size was determined based on the previous publications (Du et al., 2018, Science)<br>The chosen sample size was sufficient to determine statistical significance in our experiments.
Data exclusions	No data were excluded from the analysis.
Replication	All data were repeatable, with the number of replicates provided in text and/ or figure legends.
Randomization	Allocation of test plants used in our study was random. There was no targeted selection of individual plants for specific treatments.
Blinding	Blinding was not relevant to our study as we performed experiments in plant and it does not include clinical trials. In plant, biology blinded/ double-blinded studies are uncommon.

Reporting for specific materials, systems and methods

We require information from authors about some types of materials, experimental systems and methods used in many studies. Here, indicate whether each material, system or method listed is relevant to your study. If you are not sure if a list item applies to your research, read the appropriate section before selecting a response.

Materials & experimental systems

n/a	Involved in the study
☐	☒ Antibodies
☐	☒ Eukaryotic cell lines
☒	☐ Palaeontology and archaeology
☒	☐ Animals and other organisms
☒	☐ Clinical data
☒	☐ Dual use research of concern
☒	☐ Plants

Methods

n/a	Involved in the study
☒	☐ ChIP-seq
☒	☐ Flow cytometry
☒	☐ MRI-based neuroimaging

Antibodies

Antibodies used	anti-GFP antibody (Takara clonteck, 632381), 1:5000 dilution,<br>anti-mCherry antibody (Agrisera AS184179), 1;2000 dilution,<br>polyclonal goat anti-mouse IgG-HRP (Santa Cruz Biotechnology, sc2005), 1:5000 dilution.
Validation	anti-GFP antibody (Takara clonteck, 632381),https://www.takarabio.com/products/antibodies-and-elisa/fluorescent-protein-antibodies/green-fluorescent-protein-antibodies?catalog=632381<br>anti-mCherry antibody (Agrisera AS184179), https://www.agrisera.com/en/artiklar/mcherry-affinity-purified.html<br>polyclonal goat anti-mouse IgG-HRP (Santa Cruz Biotechnology, sc2005), https://www.scbt.com/p/goat-anti-mouse-igg-hrp

Eukaryotic cell lines

Policy information about cell lines and Sex and Gender in Research

Cell line source(s)	Sf21 insect cell line (Invitrogen)
Authentication	none of the cell lines were authenticated
Mycoplasma contamination	cell line was not tested for mycoplasma contamination
Commonly misidentified lines<br>(See ICLAC register)	no commonly misidentified cell lines were used in this study
